# CITEMO^XMBD^: A flexible single-cell multimodal omics analysis framework to reveal the heterogeneity of immune cells

**DOI:** 10.1080/15476286.2022.2027151

**Published:** 2022-02-07

**Authors:** Huan Hu, Ruiqi Liu, Chunlin Zhao, Yuer Lu, Yichun Xiong, Lingling Chen, Jun Jin, Yunlong Ma, Jianzhong Su, Zhengquan Yu, Feng Cheng, Fangfu Ye, Liyu Liu, Qi Zhao, Jianwei Shuai

**Affiliations:** a Department of Physics, And Fujian Provincial Key Laboratory for Soft Functional Materials Research, Xiamen University, Xiamen, China; b National Institute for Data Science in Health and Medicine, and State Key Laboratory of Cellular Stress Biology, Innovation Center for Cell Signaling Network, Xiamen University, Xiamen, China; c Wenzhou Institute, University of Chinese Academy of Sciences, and Oujiang Laboratory (Zhejiang Lab for Regenerative Medicine, Vision and Brain Health), Wenzhou, Zhejiang, China; d State Key Laboratories for Agrobiotechnology, Department of Nutrition and Health, College of Biological Sciences, China Agricultural University, Beijing, China; e School of Life Sciences, Xiamen University, Xiamen, China; f Institute of Biomedical Big Data, School of Ophthalmology & Optometry and Eye Hospital, School of Biomedical Engineering, Wenzhou Medical University, Wenzhou, China; g Beijing National Laboratory for Condensed Matter Physics and Laboratory of Soft Matter and Biological Physics, Institute of Physics, Chinese Academy of Sciences, Beijing, China; h School of Physical Sciences, University of Chinese Academy of Sciences, Beijing, China; i Chongqing Key Laboratory of Soft Condensed Matter Physics and Smart Materials, College of Physics, Chongqing University, Chongqing, China; j School of Computer Science and Software Engineering, University of Science and Technology Liaoning, Anshan, China

**Keywords:** CITE-seq, multi-omics, data integration, single-cell, immune system

## Abstract

Simultaneous measurement of multiple modalities in single-cell analysis, represented by CITE-seq, is a promising approach to link transcriptional changes to cellular phenotype and function, requiring new computational methods to define cellular subtypes and states based on multiple data types. Here, we design a flexible single-cell multimodal analysis framework, called CITEMO, to integrate the transcriptome and antibody-derived tags (ADT) data to capture cell heterogeneity from the multi omics perspective. CITEMO uses Principal Component Analysis (PCA) to obtain a low-dimensional representation of the transcriptome and ADT, respectively, and then employs PCA again to integrate these low-dimensional multimodal data for downstream analysis. To investigate the effectiveness of the CITEMO framework, we apply CITEMO to analyse the cell subtypes of Cord Blood Mononuclear Cells (CBMC) samples. Results show that the CITEMO framework can comprehensively analyse single-cell multimodal samples and accurately identify cell subtypes. Besides, we find some specific immune cells that co-express multiple ADT markers. To better describe the co-expression phenomenon, we introduce the co-expression entropy to measure the heterogeneous distribution of the ADT combinations. To further validate the robustness of the CITEMO framework, we analyse Human Bone Marrow Cell (HBMC) samples and identify different states of the same cell type. CITEMO has an excellent performance in identifying cell subtypes and states for multimodal omics data. We suggest that the flexible design idea of CITEMO can be an inspiration for other single-cell multimodal tasks. The complete source code and dataset of the CITEMO framework can be obtained from https://github.com/studentiz/CITEMO.

## Introduction

Many types of single-cell sequencing technologies have been proposed with the development of molecular biology, microfluidics, and nanotechnology [1,2]. The existing single-cell sequencing experimental technology focuses on the measurement of independent modalities, especially the transcriptome. Single-cell transcriptome sequencing has been developed with many powerful analytical methods, which are widely used in cell type identification [3–6], trajectory inference [7–10], regulatory network inference [11–14], single-cell transcriptome dynamics analysis [15,16], etc [17]. These analysis methods based on the independent modalities have promoted our understanding of cellular diversity and developmental landscapes [18–21].

Nowadays, it is more interesting to detect and analyse multimodal omics simultaneously in individual cells to build a more comprehensive molecular view of cells [22–26]. For example, in 2017, CITE-seq was proposed, which can simultaneously measure single-cell transcriptome and cell-specific protein data [27,28]. In the same year, REAP-seq was introduced, which is similar to CITE-seq, using oligonucleotide cross-linked antibodies to detect cell protein and transcript levels [29]. These two technologies have similar principles. They capture the transcriptome while capturing Antibody-Derived-Tags (ADT) to count proteins. Other technologies, such as PLAYR (proximity ligation assay for RNA), can also detect the expression level of specific proteins at the single-cell level [30–34]. Compared with other technologies, CITE-Seq and REAP-seq have mature commercial solutions, and they are one of the most popular single-cell multimodal omics technologies. Since the multimodal omics data format of REAP-seq and CITE-seq are similar and their analysis procedures are also similar, the following will use CITE-seq to collectively refer to these two technologies.

Several single-cell multimodal analysis methods have been proposed for the CITE-seq technique by now. In 2018, Satija Lab launched Seurat v3, which can analyse the transcriptome and ADTs data separately but could not integrate them [3]. Later, the updated Seurat v4 introduced the weighted-nearest neighbour analysis that sets the weights for transcriptome and ADT respectively and then constructed a weighted nearest neighbour graph to integrate these modalities [6]. In 2021, Gayoso et al. developed totalVI based on the deep learning to construct two variational autoencoders for transcriptomic data and ADT data, respectively, in which the two autoencoders share their mean parameters as an integrated characterization of multimodal omics for downstream analyses such as cell clustering [35].

There remain several challenges for multimodal omics analysis, although there are a few methods for analysing the multimodal omics data. First, the process of preprocessing and integrating multimodal omics data may introduce false signals [36]-^38^. Secondly, transcriptome and ADT data have different biological properties and functions, and the analysis process of multimodal omics should retain their characteristics [2,22–24,26,37]. Finally, the analysis results of multimodal omics should be able to correspond to the analysis results of independent modalities.

Here, we design a flexible framework, CITEMO, to comprehensively explore the single-cell multimodal omics. CITEMO framework covers a series of processes designed for multimodal data and simultaneously outputs the transcriptome, ADT and multimodal omics analysis results. Using CITEMO, we perform a multimodal analysis on a dataset of Cord Blood Mononuclear Cells (CBMC) which was annotated by previous work [27]. The outcomes indicate that our multimodal omics integrated analysis method can identify cell subtypes and have a good correspondence with independent modalities. In addition, we find some special immune cells that express several different types of immune cell markers and propose co-expression entropy to analyse them. To further validate the robustness of CITEMO, we analyse the Human Bone Marrow Cell (HBMC) samples [3] and further identify the different states of the same cell type. In short, CITEMO framework is an excellent flexible analysis method for single-cell multimodal omics, which can accurately identify the cell subtypes and states.

## Results

### Flexible multimodal omics analysis framework

**Figure 1.**
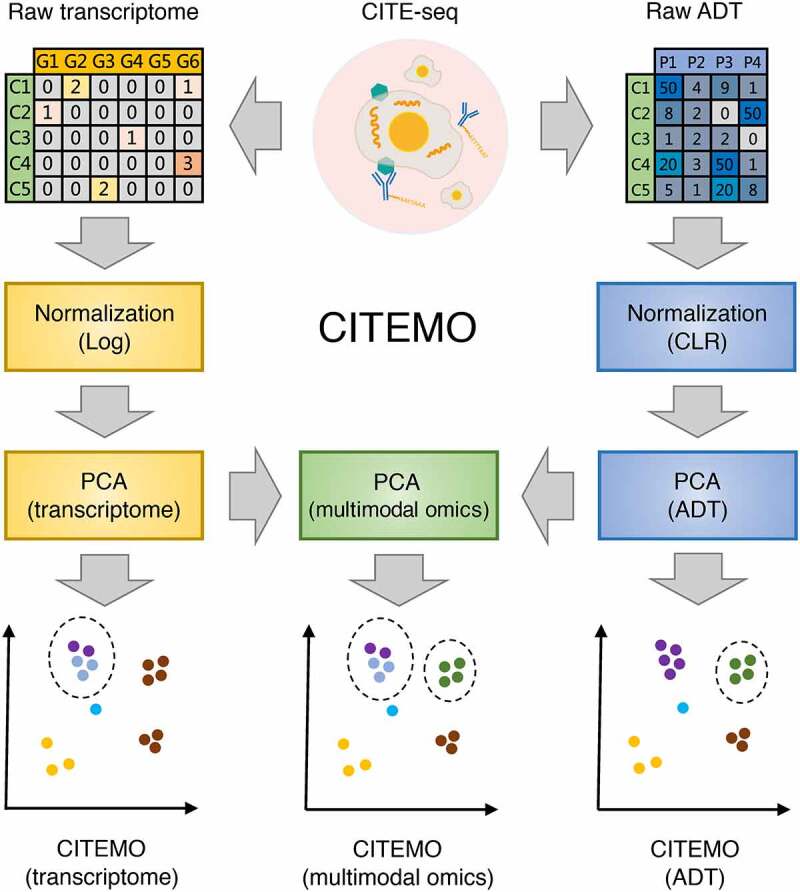
The workflow of CITEMO framework. The multimodal omics data obtained from the experiment are divided into raw transcriptome and raw ADT. They are normalized after preliminary quality control, and then applied PCA dimensionality reduction, respectively. On the one hand, the low-dimensional representations of the transcriptome and ADT are used for clustering. On the other hand, they are used for multimodal omics clustering by PCA dimensionality reduction. Finally, the clusters of transcriptome, ADT and multimodal omics are visualized using UMAP of multimodal omics.

The CITEMO framework consists of three main steps (Fig. 1). First, in the preprocessing stage, we simultaneously extract the raw transcriptome and the raw ADT data from the CITE-seq experiment, separately. After quality control, the remained raw transcriptomic data and raw ADT data are performed preprocessing, separately. We perform logarithm normalization on transcriptome data, while apply the centred logarithmic ratio (CLR) algorithm to normalize ADT data. Subsequently, the transcriptome and the ADT data are rescaled to the range of 0 to 1 by MinMaxScale, respectively.

Then, we consider dimensionality reduction to obtain the low-dimensional representations. Because of the huge difference in the feature dimensions of transcriptome and ADT data, compressing their features to similar dimensions can eliminate the influence of the difference in feature dimensions for downstream analysis. For example, the transcriptome modality has more than 10,000 features, but the ADT modality only has less than 500 features. We use Principal Component Analysis (PCA) to reduce their features to similar sizes. After dimensionality reduction, the transcriptome data and ADT data are transformed into low-dimensional transcriptome representation and low-dimensional ADT representation, respectively, which represent the relative utility of independent modalities. Subsequently, the low-dimensional transcriptome representation and the low-dimensional ADT representation are scaled to the range of 0 to 1 by MinMaxScale, respectively. Then these low-dimensional representations are integrated by PCA again to obtain low-dimensional multimodal representations. It is worth noting that the algorithms for dimensionality reduction and data integration in the CITEMO framework are all PCA. This strategy of using the same algorithm as much as possible between different modal data can further avoid the introduction of the error caused by algorithm differences.

Next, we perform Leiden clustering algorithm for the low-dimensional representations of transcriptome, ADT and multimodal omics, respectively. Finally, we use the low-dimensional representation of multimodal omics to generate uniform manifold approximation and projection (UMAP) visualizations. Such UMAP of multimodal omics is also applied to the transcriptome and ADT data to visualize the position of cells in a two-dimensional plane. Although transcriptome, ADT and multimodal omics have the same UMAP visualization, they still retain their respective cell subpopulations and are represented by different colours. By sharing the same UMAP visualizations, the cell subpopulations between different modalities can be compared easily. With these analysing steps, the CITEMO framework can capture the heterogeneous states of cells at the levels of transcriptome, ADT and multimodality simultaneously.

### Cell subtypes of multimodal omics data captured by CITEMO

**Figure 2.**
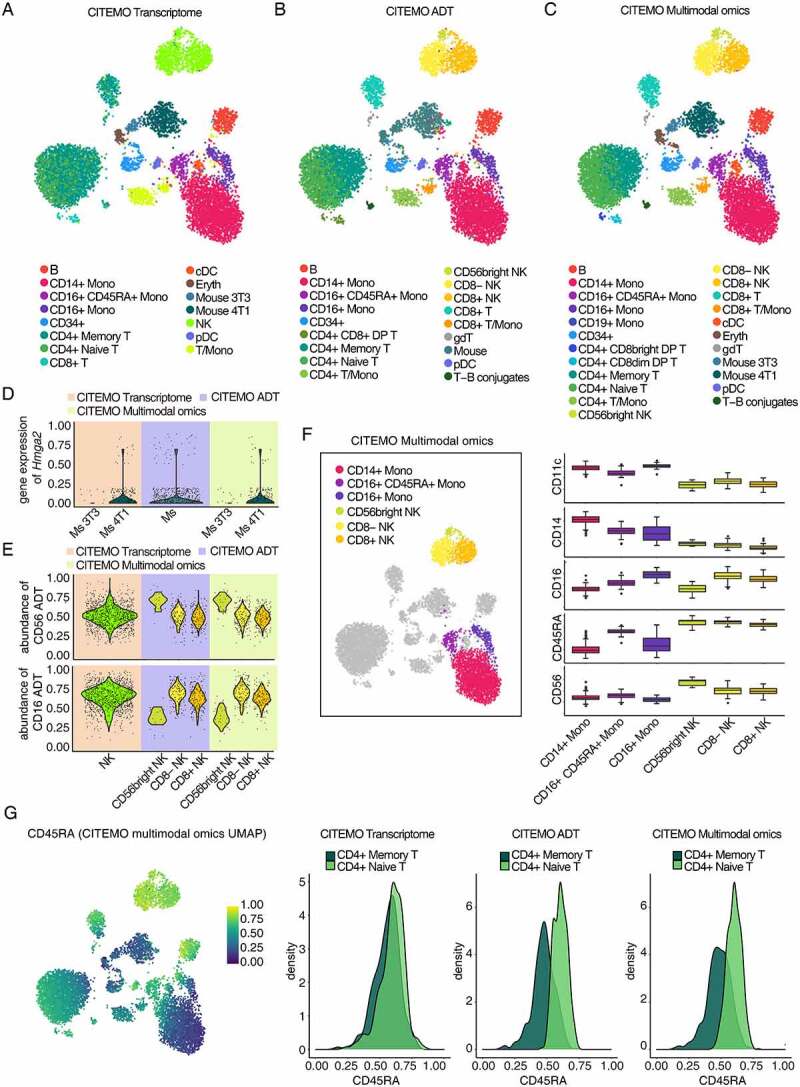
Characterizing heterogeneity with CBMC sample. (A-C) UMAP visualizations of clustering results. The annotation of cell clusters was analysed by CITEMO using transcriptome modality (A), ADT modality (B) and multimodal omics (C) data. (D) Violin plots of Hmga2 gene expression in mouse cell clusters. (E) Violin plots of CD56 (up) and CD16 (down) ADT abundance in NK cell clusters. Different background colours in (D) and (E) indicate that different modalities were used for clustering analysis by CITEMO. (F) The clustering results of NK cells and Monocytes obtained by CITEMO multimodal omics on the left, and the box plots on the right showing the different ADT abundance of the NK cells markers of CD56 and CD16, the Monocytes markers of CD11c and CD14, and a proliferating marker CD45RA in six distinct clusters. (G) A feature plot of CD45RA ADT abundance with CITEMO multimodal omics UMAP shown on the left, and the density distributions of CD45RA ADT in CD4+ Memory T cells and CD4+ Naïve T cells under the indicated modalities given on the right.

To investigate the performance of the above pipeline, we analyse the CBMC sample sequenced by Stoeckius et.al. [27]. In this dataset, the small quantities of 3T3 and 4T1 mouse cells are mixed into CBMC samples to assess the sensitivity of the CITE-seq technique [27]. We simultaneously identify cell populations from the perspectives of transcriptome, ADT and multimodal omics, and find that all the three perspectives are able to reveal the heterogeneity of CBMC sample (Figs. 2A-2C).

**Figure 3.**
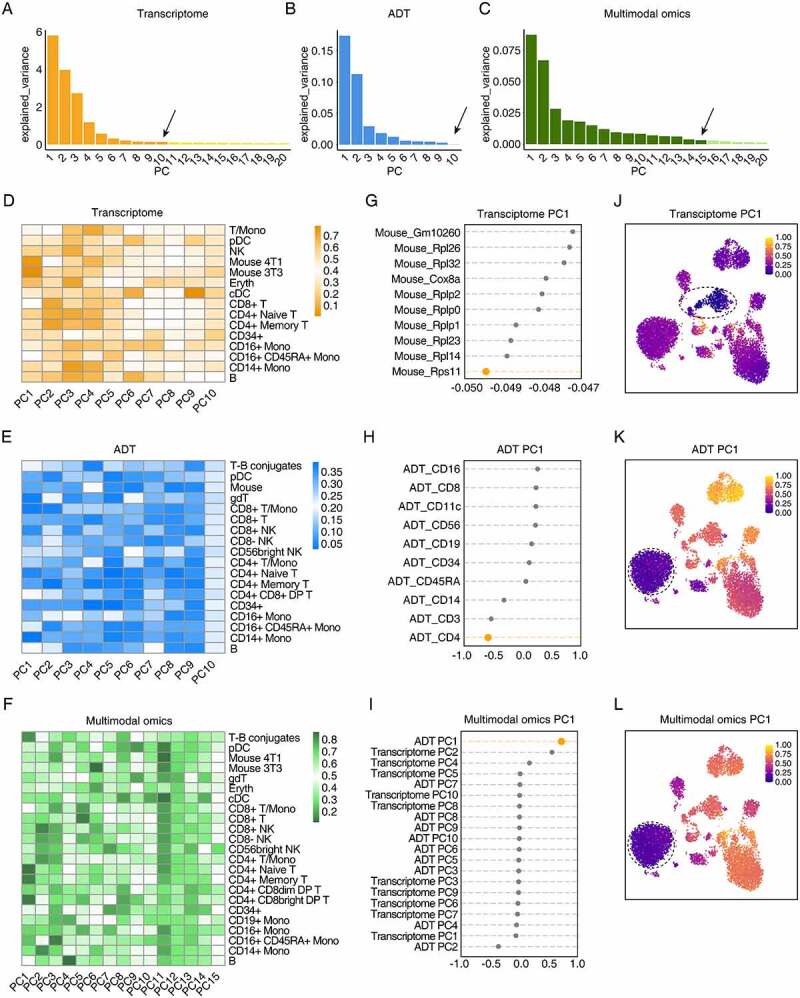
The low-dimensional representations of the heterogeneity in the CBMC sample. (A-C) The variance of the transcriptome principal components (A), the ADT principal components (B), and the multimodal omics principal components (C) explained by each selected principal component. The variance estimation uses n_samples-1 degree of freedom. The arrows indicate the reduced dimension set by the elbow method. (D-F) Heat maps of cell clusters and selected principal components. The average value of each principal component of the cell clusters divided by transcriptome (D), ADT (E) and multimodal omics (F). (G-I) The projections of the features along the principal component PC1 direction sorted from the small to the large PC value for the gene in the transcriptome (G), the ADT (H), and the transcriptome PC and ADT PC in the multimodal omics analysis (I). (J-L) UMAP visualization of principal component PC1 for transcriptome (J), ADT (K) and multimodal omics (L), respectively. In (J-L) the circles with dashed line indicate mouse cell clusters (J) and CD4 + T cell clusters (K&H), respectively.

First, the transcriptome and ADT data are separately performed PCA to obtain the low-dimensional representation. We use the elbow method to set the PCA parameters. In this study, the top 10 principal components (PC) are selected as the low-dimensional representation for CITEMO transcriptome data (Fig. 3A). Since only 10 ADTs are involved in the study after quality control, all of 10 ADTs are considered as the low-dimensional representation for CITEMO ADTs (Fig. 3B).

Next, the cell clustering process is performed for the low-dimensional representations of the transcriptome and ADT data, respectively. Then the cell types are manually annotated based on the cell markers (Supplementary Figure 1A&1B). CITEMO transcriptome and CITEMO ADT analysis identify most of the known CBMC cell types consistently, leaving several differently annotated cell subtypes (Fig. 2A, Supplementary Figures 1a&1b&2a&2b). For example, the CITEMO transcriptome successfully identifies the mixed mouse cells in CBMC samples, while the CITEMO ADT could not detect these mouse cells (Fig. 2D). The CITEMO transcriptome further divides the mouse cells into 3T3 and 4T1 cells according to the gene expression of *Hmga2* (Fig. 2D) [27]. Since the abundance of mouse-associated ADT is not measured in CBMC samples, the mouse cells could not be identified by ADT information alone.

At the same time, relying only on genetic information may also miss some important cell subtypes. For example, only a population of NK cells are found with the transcriptome analysis, while the ADT analysis successfully identifies three subtypes of NK cells, including CD56bright NK [38–40], CD8- NK [41,42] and CD8+ NK [42–44] (Fig. 2E and Supplementary Figure 1A). These three subtypes are also supported by the previous studies [45].

The different recognition ability of cell subtypes for transcriptome and ADT analysis is caused by the difference in PCA analysis. We use PCA to convert the expression of genes and ADT into PCs. The distributions of PCs of cell subtypes in transcriptome and ADT modalities are specific, which implies that PCs represent specific information about cell subtypes (Figs. 3D&3E). For example, PC1 in the transcriptome modality is closely related to mouse genes (Figs. 3G&3 J), while the distribution of PC1 in ADT mode is consistent with the distribution of CD4 + T cells (Figs. 3H&3 K).

Integrating the low-dimensional representation of the transcriptome and ADT data can obtain a multimodal omics representation, giving a more comprehensive characterization of the heterogeneity of cells. To avoid the error introduced by different algorithms, we keep using the PCA algorithm to integrate multimodal omics. We apply the elbow method to select the first 15 PCs as the low-dimensional representation of multimodal omics (Fig. 3C). Then, the cell clustering process is performed for the low-dimensional representation of multimodal omics, followed by the manual annotation of cell types. The results given by the CITEMO multimodal omics cover all cell subtypes identified by CITEMO transcriptome and CITEMO ADT separately (Fig. 2C, Supplementary Figures 1A&2C). CITEMO multimodal omics successfully identifies 3T3 and 4T1 mouse cells, as given by CITEMO transcriptome (Fig. 2D). Moreover, CITEMO multimodal omics also successfully identifies three subtypes of NK cells (Fig. 2E and Supplementary Figure 1A), as given by CITEMO ADT.

Another noteworthy finding is that CITEMO multimodal omics identified CD16+ CD45RA+ monocyte which was annotated as NK cells by a previous study (Fig. 2F) [46,47]. CD16+ CD45RA+ monocytes are similar to some NK cells, in that they all express CD16 (Fig. 2F and Supplementary Figure 1A). This may be the reason why it was identified as NK cells by previous methods [3]. However, CD16+ CD45RA+ expresses CD14 and CD11c, which are markers of monocytes (Fig. 2F and Supplementary Figure 1A) [48]. Therefore, we believe that it is not an NK cell but a special type of monocyte. Alternatively, CD16+ CD45RA+ monocytes may be the activated monocytes due to their higher CD45RA expression than other types of monocytes (Fig. 2F). This implies that CITEMO framework can detect the cell states.

We further compare the results given by CITEMO multimodal omics with previous studies [3]. CITEMO multimodal omics identifies naive CD4 T cells and memory CD4 T cells from the CD4 T cells according to the abundance of CD45RA ADT (Fig. 2G and Supplementary Figure 1A) [49,50]. The CITEMO ADT also identifies two CD4 T cell subtypes, i.e. native CD4 T and memory CD4 T (Fig. 2G). As a comparison, the CITEMO transcriptome fails to distinguish the naive CD4 T cells from memory CD4 T cells, which is similar to the annotation of CBMC cells in previous study [3]. In summary, the CITEMO multimodal omics shows an ability to identify more cell subtypes than previous methods [3].

Each PC of CITEMO multimodal omics also shows a specific distribution in the cell clusters. For example, multimodal omics has only 10 PCs, among which PC1 is closely related to ADT PC1 (Fig. 3I), and is closely related to CD4 cells, resulting in the distribution of multimodal omics PC1 closely related to CD4 + T cells (Fig. 3L).

### Discovery of immune cell units with co-expression entropy

**Figure 4.**
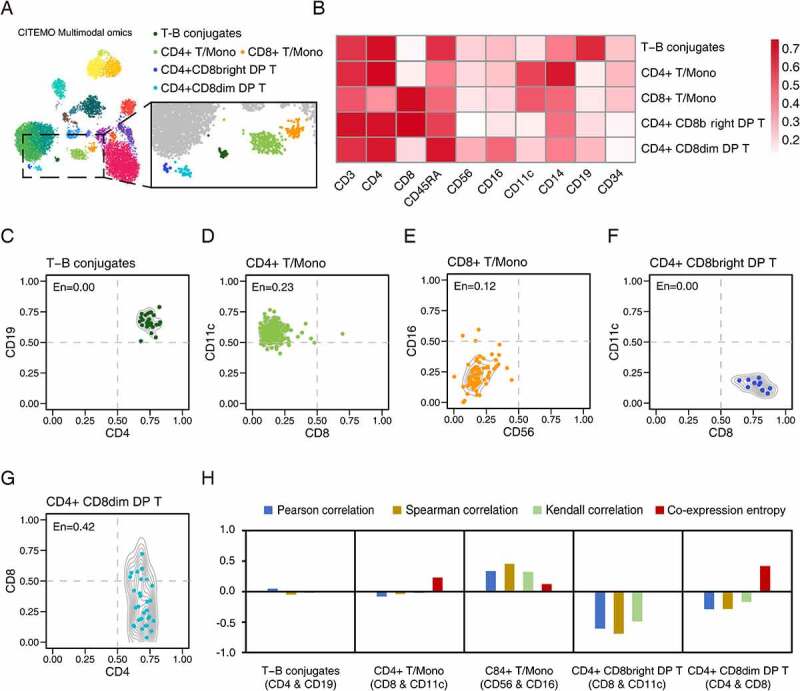
Analysis of potential co-expression cells in CBMC samples. (A) Feature plot with colour dots showing the indicated cell clusters with special co-expression. (B) Heat map of cell clusters with expressions of specific ADTs. (C-G) The ADT co-expression of CD4 and CD19 in T-B conjugates cluster (C), CD8 and CD11c in CD4 + T/Mono cluster (D), CD56 and CD16 in CD8 + T/Mono cluster (E), CD8 and CD11c in CD4+ CD8bright DP T cluster (F), and CD4 and CD8 in CD4+ CD8dim DP T cluster (G), respectively. The entropy (En) value shown in the top left corner of each figure is the corresponding co-expression entropy for each cell cluster. (H) The comparison of the evaluations of ADT combinations given by different methods, including Pearson correlation, Spearman correlation, Kendall correlation and the co-expression entropy, for cell clusters in (C-G) .

We find some interesting immune cells in the CBMC samples, and they are detected to express multiple types of immune cell markers on the cell surface (Fig. 4A and Fig. 4B). We propose four-quadrant probabilities to analyse the combination distribution of cell clusters in the 2-dimensional ADT plane. In addition, we define the co-expression entropy to detect potential co-expressed ADT combinations for clustered cells with high throughput.

Using the quadrant probability, we observe a cluster of cells that highly express CD4 (a marker of T cells) and CD19 (a marker of B cells) (Fig. 4C). Such cells with the double-positive combination of CD4 and CD19 are called T-B conjugates [51–54]. It has been reported that T-B conjugates exist in peripheral blood and are necessary for follicular helper T cells development in germinal centres [54]. As a comparison, the previous methods could not detect T-B conjugates in CBMC samples, indicating that CITEMO has more sensitive detection capabilities [3]. More examples of double-positive express of ADTs are shown in Supplementary Figure 3A.

In addition to double-positive expression, the quadrant probability also covers the combination of single-positive/single-negative or double-negative expression. For example, we find two types of T/Mono cells in CBMC, among which CD4 + T/Mono cells have a low level of CD8 (CD8 + T cell marker) and a high level of CD11c (monocyte marker) (Fig. 4D), and CD8 + T/Mono cells have double-negative expressions of CD56 and CD16 (Fig. 4E) [55]. More examples of single-positive /single-negative express of ADTs are shown in Supplementary Figure 3B, while more examples of double-negative express of ADTs are shown in Supplementary Figure 3C.

It is worth noting that some ADT combinations may span multiple quadrants. For example, for the two types of double-positive T cells (DP T) found in CBMC samples, the CD4+ CD8bright DP T highly expresses CD8 (Fig. 4F and Supplementary Figure 3A), while the CD4+ CD8dim DP T expresses CD8 widely with a small percentage of cells showing a relatively high expression of CD8 and most cells expressing CD8 at low levels (Fig. 4G and Supplementary Figures 3B, 3D). The expression of CD8 in this state was defined as dim by previous work [56,57]. The DP T cells are reported in the blood and peripheral lymph tissues of many species and presented at the T cell developmental stage [56–59].

A discussion of quadrant probabilities for ADT co-expression distributions of cells can quantitatively reveal the heterogeneity of cells. But there are many ADT combination modes in each cell cluster, resulting in a hardly direct-viewing presentation of co-expression distributions for high throughput data mining. A common solution is to calculate the correlation coefficient of the ADT combination. However, we find that the common correlation coefficients, including Pearson correlation, Spearman correlation, and Kendall correlation, cannot characterize the distribution of ADT combinations (Fig. 4H and Supplementary Table 1).

To quantitatively measure the distribution of ADT combinations, we introduce the co-expression entropy based on the four-quadrant probabilities. The value of the co-expression entropy ranges from 0 to 1. The co-expression entropy close to 0 indicates that the majority of cells are only in one quadrant. For example, the co-expression entropy of CD4 and CD19 in T-B conjugates cells is 0 (Fig. 4H and Supplementary Table 1), because all T-B aggregates cells are distributed in the double-positive quadrant with high expression of CD4 and CD19 (Fig. 4C).

A common case is that the co-expression entropy of ADT combinations in cell clusters is relatively small but not zero and may span multiple quadrants. For example, although the CD8 and CD11c combination of CD4 + T/Mono cells spans three quadrants, the majority of CD4 + T/Mono cells are in the CD11c single-positive/CD8 single-negative quadrant (Fig. 4D). Here, the co-expression entropy of the combination of CD8 and CD11c in CD4 + T/Mono cells is 0.23 (Fig. 4H and Supplementary Table 1).

Another notable value of co-expression entropy is 0.5. A co-expression entropy around 0.5 means that the cells in the ADT combination may span mainly at 2 quadrants, possibly indicating a special biological significance. For example, in the ADT combination of CD4+ CD8dim DP T, the entropy of CD4 and CD8 is 0.42 (Figs. 4G, 4 H and Supplementary Table 1), and the entropy of CD3 and CD8 is 0.62 (Supplementary Figure 3D). According to our experience, the entropy value above 0.75 implies that it is difficult to discover the potential biological significance of the ADT combination. In summary, compared with the common correlation coefficients, the co-expression entropy can intuitively characterize the co-expression distribution of ADT combinations in a single quadrant.

### Cell states characterized by CITEMO multimodal omics

**Figure 5.**
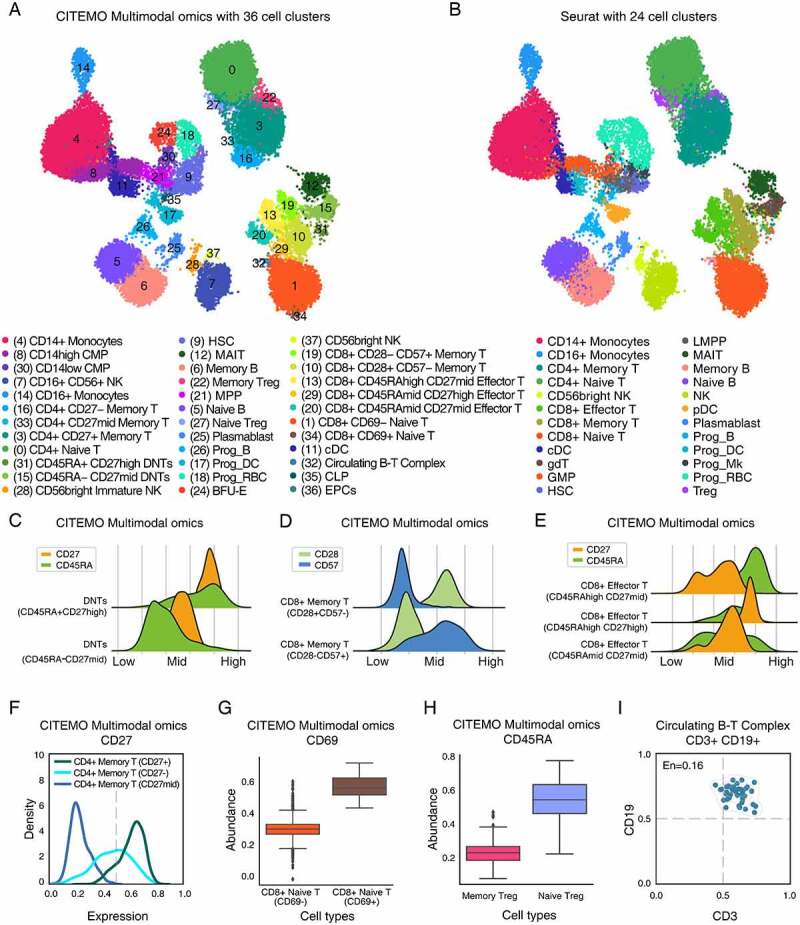
Multimodal omics analysis of HBMC samples. (A, B) UMAP visualizations of clustering results in HBMC sample with annotation of cell clusters analysed by CITEMO (A) and Seurat v4 (B), respectively. (C) The ADT abundance distributions of CD27 (Orange) and CD45RA (green) for DNT cells, giving two different states of DNT cells identified. (D) The ADT abundance distributions of CD28 (light green) and CD57 (blue) for CD8+ Memory T cells, giving two different states of CD8+ Memory T cells identified. (E) The ADT abundance distributions of CD27 (Orange) and CD45RA (green) for CD8+ Effector T cells, given three different states of CD8+ Effector T cells identified. (F) Different abundance distributions of CD27 ADT in CD4+ Memory T cells. (G) The box plots of different abundances of CD69 ADT in CD8+ Naive T cells, and (H) the box plots of the different abundances of CD45RA ADT in Treg cells, indicating the different cell subtypes. (I) Co-expression of CD3 and CD19 in Circulating B-T complex with the co-expression entropy of En = 0.16.

To further examine the robustness of CITEMO multimodal omics in big samples, we analyse the Human Bone Marrow Cell (HBMC) sample sequenced by Stuart et.al [3]. CITEMO and Seurat use different strategies to integrate multimodal data. For the transcriptome and ADTs, Seurat selected 30 and 18 PCs, respectively. In order to strictly compare the differences between CITEMO and Seurat in integrating multimodal data strategies, we also select 30 and 18 PCs for the transcriptome and ADTs, respectively. These PCs are integrated as multi-modal data by CITEMO. We apply Leiden to multimodal data to generate 38 cell clusters. Then, we carry out differential analysis of transcriptome (Supplementary Table 2) and ADTs (Supplementary Table 3) on these 38 cell clusters. After performing a manual merge of the two closest clusters (Cluster2/4 and Cluster3/23), we identify 36 distinctive clusters based on the multimodal dataset (Fig. 5A, Supplementary Figures 4A&4B&5A&5B). The annotation of HBMC cells given by CITEMO framework is similar to that analysed by Seurat v4 (Fig. 5B) [6].

It is worth noting that the clustering method of CITEMO multimodal omics enables a detailed description of the differentiation and activation states of cells based on proteins (CD27, CD45RA, CD69, CD28 and so on). For example, cluster 31 and cluster 15 were annotated by Seurat as gamma delta T (gdT) cells (Figs. 5A&5B). Considering that gdT is abundant in the intestinal mucosa, they are also present in the thymus, peripheral lymphoid tissue and peritoneum, but the content in the bone marrow is limited under physiological conditions [60]. According to the expression of CD4 and CD8, cluster 31 and cluster 15 are considered to be CD3+ CD4-CD8- T cells (double-negative T cells, DNTs) (Supplementary Figures 4B&5A) [61]. According to the difference between CD45RA and CD27, they are identified as DNTs (CD45RA-CD27mid) and DNTs (CD45RA+CD27high) (Fig. 5C). Their differences are mainly reflected in the degree of differentiation and activation.

Another example is that CITEMO multimodal omics identifies three states of effector CD8 + T cells based on the changes of CD45RA and CD27 (Fig. 5D, Supplementary Figures 4B&5A) [62,63]. CD45RAhighCD27mid, CD45RAmidCD27mid, CD45RAmidCD27high mean that these three groups of cells are at different activation levels. This division plays a certain auxiliary role in analysing how effector CD8 + T cells exert their physiological functions. Similarly, clusters 13, 20, and 29 are identified as CD8+ effector T cells by Seurat (Figs. 5A&5B). CITEMO multimodal omics classifies that cluster 13 is CD45RAhighCD27mid, cluster 20 is CD45RAmidCD27mid, and cluster 29 is CD45RAmidCD27high, indicating that these three groups of cells are at different activation levels (Fig. 5E, Supplementary Figures 4B&5A). Compared with Seurat, CITEMO multimodal omics further recognizes the status of CD8+ effector T cells, which may help to understand how effector CD8 + T cells exert their physiological functions and play a certain auxiliary role.

CD27 is also a marker of CD4 + T cell activation. According to the expression of CD27, memory CD4 + T cells are further divided into three states: CD4+ memory T (CD27+), CD4+ memory T (CD27-) and CD4+ memory T (CD27mid) (Fig. 5F, Supplementary Figures 4B&5A) [64–66]. The last example of cell state is CD69. As a signal transduction receptor, CD69 participates in the early activation of T cells, natural killer cells, monocytes and platelets [67]. CD8+ naive T are divided into CD8+ naive T (CD69-) and CD8+ naive T (CD69+) by CITEMO multimodal omics according to the different expressions of CD69 (Fig. 5G, Supplementary Figures 4B&5A) [68]. This classification allows a more efficient differentiation of different activation states of immune cells and thus a more detailed description of human immunity.

It is worth noting that CITEMO multimodal omics can discover new cell subtypes and recognize the cell states. Both Seurat and CITEMO multimodal omics identify Treg cells [69]. However, different to Seurat, CITEMO multimodal omics can further divide CD45RA into memory Treg and naïve Treg (Fig. 5H, Supplementary Figures 4B&5A) [70]. We also find the circulating B-T complex in HBMC samples (Fig. 5I, Supplementary Figures 4B&5A). These results suggest that CITEMO multimodal omics can better recognize the cell states of cell subtypes than Seurat.

### The integrating utility of CITEMO multimodal omics

The utility of CITEMO multimodal omics is essentially derived from its low-dimensional representation with PCA, which is closely related to the distribution of cell clusters (Supplementary Figure 6A&6B). For HBMC data, we select the top 30 PCs to represent transcriptome data and the top 18 PCs to represent ADT data. The PC of multimodal omics comes from the integrated projection of these 30 transcriptome PCs and 18 ADT PCs in the principal component direction, which can represent the heterogeneity of cells [3,6].

To analyse the relationship between the feature and the PCs of multimodal omics, we select the feature value with the largest variance among all cells as the representative of the feature. We find that the contribution of the first 18 PCs of multimodal omics mainly comes from ADT PC, and the contribution of the following 12 PCs of multimodal omics mainly comes from transcriptome PC (Supplementary Figure 6A).

In PCA transformation, the higher order of the PC means the stronger ability to represent features. Just because the first 18 PCs of multimodal omics are closely related to ADT PCs, CITEMO multimodal omics can identify ADT-related cell states more accurately than previous studies [3,6]. Although ADT PCs are closely related to the top-ranked PCs of multimodal omics, the number of ADT PCs is small and its ability to characterize cell heterogeneity is certainly limited. The transcriptome PC can make up for this defect by contributing to the following 12 PCs of multimodal omics PCs. In addition, we also notice that there is a weak correlation between the top-ranked transcriptome PC and ADT PC (Supplementary Figure 6C).

Furthermore, we find that the differences in cell subtype states are more pronounced at the ADT level than at the RNA level (Supplementary Figure 7A&7B). This may be due to the fact that the proteins characterized by ADT are closer to the cell phenotype than the transcriptome. Thus, ADT information in multimodal data is more suitable for characterizing the cell states, while the high latitude genetic information can identify cell subtypes. CITEMO multimodal omics combines these advantages.

Finally, we evaluate the efficiency of integrating multimodal omics data with the same preprocessing steps for CITEMO and the previous methods [6]. The PCA-based CITEMO framework shows higher running efficiency than previous methods (Supplementary Figure 8).

### Discussion

Single-cell multimodal omics technology represents an exciting frontier in single-cell sequencing. In this study, we propose a single-cell multimodal omics analysis framework, called CITEMO, to simultaneously perform single-cell analysis of transcriptomic, ADT and multimodal omics. The CITEMO framework adopts flexible design principles. We believe that excessive processing of biological information will inevitably introduce non-biological information, which will have an uncontrollable impact on downstream analysis. For example, for the same single-cell data, different algorithms may produce different results. and the CITEMO framework applies the same analysis method for different modal data, which can effectively avoid the introduction of these factors.

In the data preprocessing stage, we only use the simplest logarithmic normalization to process the transcriptome data. This strategy helps to preserve true biological differences. Similarly, considering that it is controversial whether needs to supplement single-cell transcriptome data [71], CITEMO does not have a process for supplementing transcriptome data. For ADT data, we refer to the processing method of Stoeckius et al [27]. Compared with transcriptome data, ADT data have a larger data range and have no sparsity problem. To eliminate their differences in the data range, we implement MinMaxScale to scale the transcriptome and ADT data to the range of 0 to 1, respectively. Then the transcriptome and ADT data are reduced by the PCA algorithm to obtain their low-dimensional representations, respectively. Previous studies have shown that PCA has the ability to extract features of transcriptome and ADT data [3,4,6].

In this study, we use PCA to integrate transcriptome and ADT data. According to the principle of PCA, it maps the combined features of the transcriptome and ADT to the orthogonal feature space and extracts the feature projection of the principal component direction with the greatest difference. PCA can overcome the extensive technical noise in any single feature of single-cell data. Each PC represents a collection of features. Usually, selecting more PCs means introducing more information for downstream analysis. However, selecting more PCs may also introduce noise for downstream analysis, which is contrary to our original intention of using PCA. In this study, we use the elbow method to select the number of PCs (refer to ‘Dimensionality reduction of genes’ in ‘Materials and Methods’). Both the dimensionality reduction of single-cell transcriptomics and the integration of multiple omics use the PCA algorithm to further avoid errors introduced by algorithm differences. The analysis results of CBMC samples show that the multiple omics integrated by PCA can cover the heterogeneous information of single omics. Furthermore, the analysis of HBMC samples implies that, compared to the previous methods [6], the implementation of PCA in the CITEMO framework to integrate multiple omics data can effectively capture the heterogeneity of cell states and subtypes. Moreover, the parameters of PCA are easy to set and have a wide range of practical values. In short, the CITEMO framework can successfully analyse the single-cell multimodal omics data.

It is worth noting that during the analysis of CBMC samples, we find some ADTs with specific co-expression. To better observe these specific co-expression behaviours, we propose the four-quadrant probabilities to quantitatively describe the double-positive state, the single-positive/single-negative state and the double-negative state of cell ADT distributions. With the help of quadrant probabilities, we suggest that the co-expression ADTs may play an important role in immunity and disease development.

For example, in the CBMC samples, we find a type of DP T cell, showing a co-expression of the double-positive state of CD4 and CD8. They are susceptible to viral activation and clearance and play important roles in many diseases such as HIV and cancer [72]. Recently, JG et al. found that DP cells decreased significantly with the severity of the disease for the COVID-19 patients in 4–7 days [73], which implies that DP T cells have potential to become the target in the treatment of early COVID-19 patients. Another example is about the T-B conjugates, showing a co-expression of the double-positive state of CD4 and CD19. Zhang et al. noted that the special CD4+ CD19+ conjugates are more likely to bind to the HIV-1 virus compared with the regular CD4 + T cells, and so their number and percentage both decrease with the progress of HIV-1 disease [54]. This implies that the CD4+ CD19+ conjugate cells may be one of the targets for testing and treatment of HIV-1 disease symptoms.

In addition, we introduce co-expression entropy for high throughput mining of ADT co-expression for each cluster of cells. If a cluster of cells is distributed in one quadrant only in a 2-dimensional ADT plane, the corresponding co-expression entropy is close to zero. When the cells are distributed evenly in four quadrants in a 2-dimensional ADT plane, the co-expression entropy approaches 1, implying that the division of cell clusters may be unreasonable, and there may be multiple types of cells inside the cell clusters. The application of co-expression entropy and four-quadrant probabilities can help us to quickly identify the status of the double-positive, single-positive/single-negative, or double-negative co-expression and the cell subtypes. With more ADT detected, we believe that the co-expression entropy and the quadrant probabilities may become an important index for cell subtype state recognition.

Most single-cell analysis methods only focus on the cell subtypes. However, cell subtypes do not provide a complete picture of cellular heterogeneity. Cells with the same subtype may have different cellular states, and they are closely related to cellular functions, especially in disease development. ADT can provide reliable information about the state of the cell. This implies that the ADT modality information may be more important in multimodal omics analysis. Unfortunately, the cost of measuring ADT is very high, which results in very few types of ADT information. After integrating the transcriptome and ADT modalities, CITEMO cannot only find more cell subtypes but also identify more cell states than previous methods [3].

Essentially, CITEMO maps the linearly reduced transcriptome features and linearly reduced ADT features to a new linear subspace through PCA. This method has three advantages. First, we use linear methods to extract important components between transcriptome features and ADT features, which makes the integrated multi-modal data have a better correspondence with the two types of features. Second, our integration model is adjustable and only the number of selected PCs needs to be adjusted. Finally, our method is more efficient than previous studies (Supplementary Figure 8), and can be widely used in large-scale data integration.

Although CITEMO is a powerful single-cell multimodal omics analysis framework, the current version of CITEMO is only suitable for integrating multimodal omics data collected by CITE-Seq/REAP-Seq. The analysis process of the CITEMO framework is optimized for CITE-Seq/REAP-Seq. We cannot guarantee that CITEMO is applicable to data collected by other single-cell multimodal omics experimental techniques. With the development of single-cell multimodal omics experimental technology, we will be compatible with a wider range of single-cell multimodal omics experimental technologies in the future.

### Conclusion

In short, CITEMO is a reliable single-cell multimodal omics analysis framework, which can reveal immune cell heterogeneity with wide applicability. CITEMO can be easily applied for large sample analysis with excellent robustness. With the development of multimodal omics sequencing technology, more and more multimodal omics data are accumulated, thus the CITEMO multimodal omics analysis may play an important role in future biomedical research.

### Materials and Methods

CITEMO can simultaneously output the analysis results of transcriptome, ADT and multimodal omics. First, we introduce the process of CITEMO to analyse the transcriptome and ADT data separately. Next, we explain how CITEMO integrates multimodal omics data. Finally, we describe the downstream analysis methods of single cells involved in this study.

### Datasets

In this study, two datasets, CBMC [27] and HBMC [3] are involved to evaluate the performance of CITEMO. The CBMC dataset contains 8617 cells in which the single-cell transcriptome measurements were paired with abundance estimation of 11 types of immune-related ADT. To test the sensitivity of CITE-seq technology, Stoeckius et al. mixed a small part of 3T3 and 4T1 Mouse cells in umbilical CBMC [27], which raises the difficulty for downstream analysis. Therefore, they removed these Mouse cells beforehand during the data analysis. In particular, the true single-cell raw data environment is extremely complex. Thus, it is not possible to fully guarantee that the tissue samples obtained in the experiment are pure. To further assess the ability of CITEMO, another data set, HBMC, which includes 30,672 scRNA-seq profiles and 25 antibodies from Bone Marrow [3], is also used for discussion in the paper. In addition, we download 4 multimodal omics data sets from the 10X webpage to evaluate the efficiency of integrating multimodal omics data.

### Gene screening strategy

We introduce the analysis steps of the CITEMO transcriptome by using the CBMC sample as an example. Because CBMC is mixed with mouse cells, we pre-screen the top 100 mouse genes with the largest coefficient of variation.

Due to the low mRNA copy initiation per cell of sequencing technology, the sequencing data of single-cell transcriptome usually suffer from the dropout phenomenon that many expressed mRNAs are not captured, resulting in zero or near-zero gene expression detected. Although various noise reduction methods for single-cell transcriptome data have been developed to remove the deletions, there is no guarantee that the single-cell transcriptome data reflect the true cellular state after the noise reduction [36,37,74]. Recently, Svensson et al. suggested that the excess of zero values in transcriptome data may be attributed to the biological variation rather than the technical defects [71]. The only thing that can be determined is that the highly expressed genes are relatively reliable. As a result, we consider a feature gene screening strategy by using the relatively highly expressed genes only for the downstream analysis. This strategy avoids the zero or near-zero impact and ensures the reliability of the screened genes.

In this study, we denote the transcription profile by, and define two indexes of total gene expression *T* and sparsity *SP* to measure the reliability of genes, which are given by

(1) $$M_{I*J}=\left(m_{ij}\right)$$

(2) $$T_{M_{i}}=\underset{j}{\sum }m_{ij}$$

(3) $$SP_{M_{i}}=\underset{j}{\sum }\left(1-\delta_{0,m_{ij}}\right)$$

Here as shown in Eq. 1, represents the gene-cell matrix element, in which is the value of transcription profile, also called UMI, *i* the index of the gene, and *j* the index of the cell, also called barcode. *M* represents a single-cell transcriptome matrix, which has *I* genes and *J* cells. Eq. 2 represents the total gene expression of gene i in all cells. Eq. 3 represents the sparsity *SP* of $M_{i}$, in which is Kronecker delta. In the CBMC data set, we select genes with *T* greater than 0.01*J and *SP* larger than 0.05*J for downstream analysis. In other words, these selected genes are expressed in at least 5% of cells and thus the expression level is not too low. HBMC samples are done by similar treatment with *T* greater than 0.01*J and *SP* greater than 0.01*J. These selected genes are less likely to suffer the dropout and are more reliable.

### Single-cell transcriptome normalization

In scRNA-seq, due to the limited molecular weight of the initial transcription in each cell, the capture and amplification efficiency of transcripts have technical differences, and so it is difficult to ensure a high degree of consistency in library preparation between samples. This also causes the system differences in the sequencing data of multiple samples due to different library sequencing coverage. To eliminate these discrepancies, the normalization process is carried out for the sequencing data. In this study, the following simple method is applied to perform data normalization,

(4) $$X_{RNA_{ij}}=\log\left(1+m_{ij}\right)$$

where is the normalized transcriptome data. Since m is very likely to be 0, it is necessary to add 1 to all transcriptome data for logarithm transformation.

### Range rescale of transcriptome data

The range of the normalized transcriptome data is still uncertain, which will affect the accuracy of downstream analysis. Therefore, the following rescaled process, i.e. MinMaxScaler, is applied to rescale the normalized transcriptome data to the range of 0 to 1,

(5) $$X_{Scaled\_RNA_{ij}}=\frac{X_{RNA_{ij}}-min\left(X_{RNA_{i}}\right)}{max\left(X_{RNA_{i}}\right)-min\left(X_{RNA_{i}}\right)}+min\left(X_{RNA}\right)$$

Here, denotes the rescaled value of the *i*-th gene in the *j*-th cell. $min\left(X_{RNA_{i}}\right)$ and $max\left(X_{RNA_{i}}\right)$ represent the minimum and maximum of the *i*-th gene in the $X_{RNA}$ across all cells, respectively, while $min\left(X_{RNA}\right)$ is the minimum of all the elements in $X_{RNA}$.

### Dimensionality reduction of genes

For a given transcriptome dataset, many genes do not provide useful information and mostly contain only zero counts. Even after filtering out these zero-count genes in the quality control step, the feature space of the dataset may exceed 10,000 dimensions. To reduce the computational burden of downstream analysis, to reduce noise in the data and also to facilitate data visualization, the common principal component analysis (PCA) is applied to reduce the dataset dimensionality. PCA can well explain the heterogeneity of the single-cell transcriptome [3,6,75]. The PCA-treated transcriptome data are noted as.

(6) $$X_{PCA\_RNA}=PCA\left(X_{Scaled\_RNA}\right)$$

Here, the dimension of matrix $X_{PCA\_RNA}$ is *k* by *J*, in which the parameter *k* of PCA is set by the elbow method [3]. Specifically, a histogram of the variance (also known as explained variance) of each PC is plotted. The PCs before the elbow of the histogram are considered to capture most of the biological variation signals, and they are reserved for downstream analysis. The RNA modality data $X_{PCA\_RNA}$ are applied for downstream analysis of the transcriptome, such as visualization, differential analysis, and multimodal integration.

### Processes of CITEMO ADT

Next, we introduce the analysis steps of CITEMO ADT, which are very similar to the processes of CITEMO transcriptome. The results obtained by CITE-seq also suffer from ADT pollution which is similar to that of protein fluorescence staining. In CBMC samples, three antibody-oligonucleotide conjugates of CCR7, CCR5 and CD10 did not specifically bind to proteins (i.e. no background signal threshold) [27]. For these potentially contaminated ADTs, we directly delete them from the preprocessing for CBMC samples, while we do not remove the low-quality ADT for HBMC samples.

In this study, we denote the ADT profile by $Q_{K*J}$,

(7) $$Q_{K*J}=(q_{kj})$$

where *Q* represents ADT abundance matrix with *K* types of ADT and *J* cells, $q_{kj}$ represents ADT matrix element with *q* for the abundance of ADT, *k* the index of the ADT, and *j* the index of the cells.

Then the following centred logarithmic ratio (CLR) transformation is applied to obtain the normalized ADT data $X_{ADT}$,

(8) $$X_{ADT_{k}}=\left[\ln\left(\frac{q_{k1}}{g\left(q_{k}\right)}\right),\ln\left(\frac{q_{k2}}{g\left(q_{k}\right)}\right),\ln\left(\frac{q_{k3}}{g\left(q_{k}\right)}\right),\ldots ,\ln\left(\frac{q_{kJ}}{g\left(q_{k}\right)}\right)\right]$$

which represents the CLR transformation of the *k*-th ADT with *g* the geometric mean.

Similar to the processes of the transcriptome, after ADT is normalized, the following rescale process with MinMaxScaler and PCA dimensionality reduction is also performed to obtain the heterogeneity of ADT samples,

(9) $$X_{Scaled\_ADT_{kj}}=\frac{X_{ADT_{kj}}-min\left(X_{ADT_{k}}\right)}{max\left(X_{ADT_{k}}\right)-min\left(X_{ADT_{k}}\right)}+min\left(X_{ADT}\right)$$

(10) $$X_{PCA\_ADT}=PCA\left(X_{Scaled\_ADT}\right)$$

The ADT modality data $X_{PCA\_ADT}$ are applied for downstream analysis of ADT, such as visualization, differential analysis, and multimodal integration.

### Processes of CITEMO multimodal omics

Since transcriptomic modality and ADT modality data possess different statistical and biological characteristics, we need an effective method to achieve multimodal integration.

First, in order to eliminate the difference in the data range of the transcriptome modality and ADT modality, the MinMaxScaler process is executed again on $X_{PCA\_RNA}$ and $X_{PCA\_ADT}$ respectively to obtain a new matrix $X_{Multimodal\_Omic}$ with values ranging from 0 to 1,

(11) $$X_{Multimodal\_Omic}=\left[\begin{array}{c} MinMaxScaler\left(X_{PCA\_RNA}\right)\\ MinMaxScaler\left(X_{PCA\_ADT}\right) \end{array}\right]$$

Then, the PCA dimensionality reduction operation is performed on $X_{Multimodal\_Omic}$ to obtain the heterogeneous representation $X_{PCA\_Multimodal\_Omic}$ from the perspective of multimodal omics,

(12) $$X_{PCA\_Multimodal\_Omic}=PCA\left(X_{Multimodal\_Omic}\right)$$

As a result, similar to $X_{PCA\_RNA}$, and $X_{PCA\_ADT}$, the multimodal omics $X_{PCA\_Multimodal\_Omic}$ are applied to the downstream analysis of multimodal omics.

### Single-cell visualization

For high-throughput single-cell omics data, visually displaying the characteristics of cell data is a very important task. In this study, the uniform manifold approximation and projection (UMAP) algorithm is used to visualize the distribution of single-cell data on a two-dimensional plane, which is based on the theoretical framework of Riemannian geometry and algebraic topology [76]. At present, many single-cell analysis methods apply UMAP instead of t-SNE as a new visualization choice.

### Cell clustering algorithm

Single-cell clustering is always an important work in the field of single-cell analysis, which allows us to infer the identity of cells. PhenoGraph is applied as the clustering method in CITEMO framework, which uses the Leiden algorithm as an emerging clustering method designed specifically for single-cell data [77,78]. Especially, PhenoGraph is optimized for the clusters with broken links in Leiden clustering distribution, giving a more reasonable clustering result with more subpopulations.

### Differential analysis

The null hypothesis of differential gene analysis is that the overall gene expression values of the two groups of cells have the same distribution. However, since these two clustering groups are obtained based on the clustering results of gene expression changes, their gene expression profiles must be essentially different. Then, the possible differential types of cells are judged based on the gene expression profile combining with the prior biological experience. In our study, Wilcoxon signed-rank test is used for differential gene analysis [79], which is a nonparametric test to determine whether the two dependent samples are selected from the populations in the same distribution. We also perform the same difference analysis on ADTs data. Similar to the difference analysis at the gene level, the difference analysis of ADTs can detect the type of ADT specifically expressed in each cell cluster. Through differentially expressed genes and ADT, researchers can infer the possible cell types of each cell cluster based on biological experience.

### Co-expression entropy

For a given cluster of cells obtained by CITEMO multimodal omics, the distribution of any ADT can be calculated. Because the abundance of ADT is normalized to the scale of 0 to 1, we consider 0.5 as the threshold (i.e. Θ=0.5) to distinguish the high and low ADT expression in the distribution. Practically, this threshold can be adjusted on-demand. As a result, two probabilities can be defined as follows,

(13) $$P_{H}\left(ADT\right)=\frac{N_{ADT\geq \Theta }}{N_{cluster}}$$

(14) $$P_{L}\left(ADT\right)=1-P_{H}\left(ADT\right)$$

Here $N_{cluster}$ represents the cell number in the given cluster, and $N_{ADT\geq \Theta }$ represents the cell number with a high ADT expression.

Then, the 2-dimensional distribution at the plane of any two different ADTs, e.g. ADT1 and ADT2, can be discussed for a given cluster of cells obtained by CITEMO multimodal omics. Divided by the threshold Θ, four quadrants can be defined in the 2-dimensional plane of ADT1 and ADT2. Then, for a cluster of cells distributed on such 2-dimensional plane, one can observe the proportions of the double-positive co-expression of cells with ADT1≥Θ and ADT2≥Θ in the first quadrant, the single-positives and single-negative co-expression of cells with ADT1≥Θ and ADT2<Θ, or with ADT1<Θ and ADT2≥Θ, the double-negative co-expression of cells with ADT1<Θ and ADT2<Θ in the third quadrant. Quantitatively, one can define the quadrant probabilities *P_i_* (*i* = 1,2,3,4) to represent the proportions of cells distributed in each of four quadrants. For example, the probability *P*_1_ in the first quadrant is defined as follows,

(15) $$P_{1}=\frac{N_{ADT1\geq \Theta \&ADT2\geq \Theta }}{N_{cluster}}=P_{H}\left(ADT1\right)*P_{H}\left(ADT2\right)$$

Here $N_{ADT1\geq \Theta \&ADT2\geq \Theta }$ represents the cell number in the first quadrant. Similarly, we have $P_{2}=P_{L}\left(ADT1\right)*P_{H}\left(ADT2\right)$, $P_{3}=P_{L}\left(ADT1\right)*P_{L}\left(ADT2\right)$, and $P_{4}=P_{H}\left(ADT1\right)*P_{L}\left(ADT2\right)$.

Furthermore, the following co-expression entropy can be defined,

(16) $$S=-\sum {\;}_{i=1}^{4}Plog_{4}\left(P_{i}\right)$$

In our simulation, if *P_i_* = 0, a very small number, such as 10^−6^, is considered to replace zero in order to avoid the logarithm calculation of zero. The co-expression entropy is closed to zero if a cluster of cells are distributed in one quadrant only in the 2-dimensional ADT plane, while S=1 can be obtained when the cells are distributed randomly in four quadrants.

## Supplementary Material

Supplemental Material

## Data Availability

CITEMO framework is an open-source collaborative initiative available in the GitHub repository (https://github.com/studentiz/CITEMO). The CBMC cell types annotated by Seurat can be found at the following website: https://satijalab.org/seurat/archive/v3.2/multimodal_vignette.html. The cell types of the HBMC dataset annotated by Seurat are derived from the analysis on this webpage: https://satijalab.org/seurat/articles/weighted_nearest_neighbor_analysis.html. The external data sets used to test operational efficiency come from the 10x Genomics website, and they can be obtained from our GitHub repository (https://github.com/studentiz/CITEMO/tree/main/Data/10x).
