# Supplementary material for: CITEMOXMBD: A flexible single-cell multimodal omics analysis framework to reveal the heterogeneity of immune cells
Source: RNA Biol. 2022 Feb 7;19(1):290–304. doi: 10.1080/15476286.2022.2027151 (PMC8824218; doi:10.1080/15476286.2022.2027151)
Supplement: Supplemental Material [file KRNB_A_2027151_SM3804.zip › supplementary/Supplementary Figures and Table.docx]

## Supplementary Figure


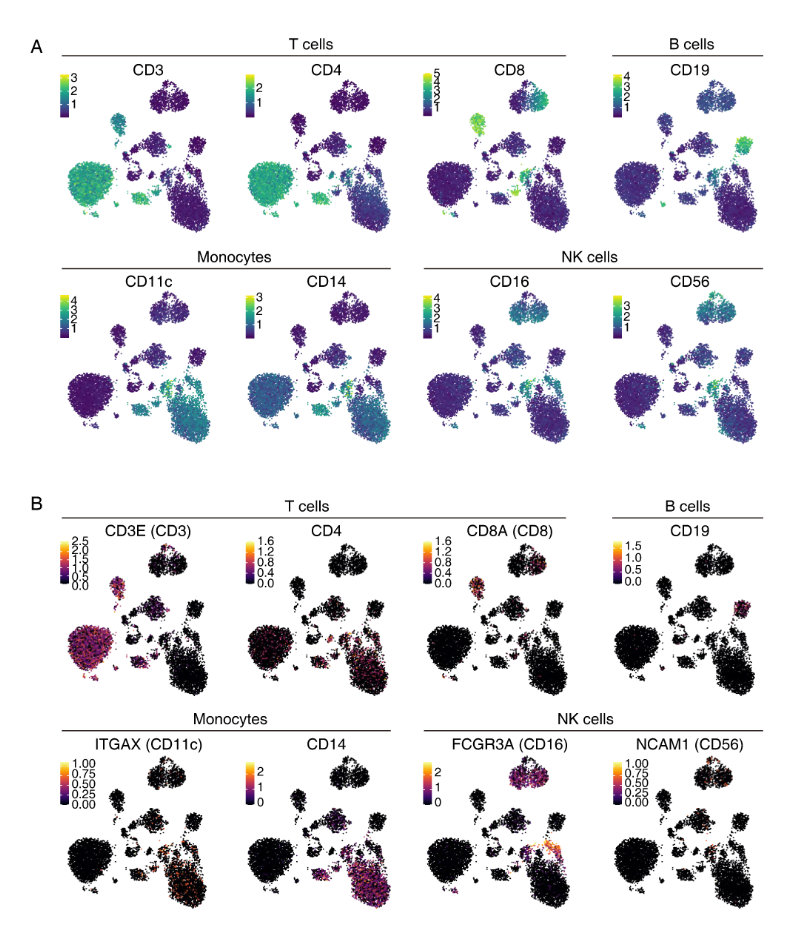


Supplementary Figure 1. Identification of different cluster in CBMC sample. Feature plots showing the ADT abundance (A) and gene expression (B) distributions of common immune cell markers in cell clusters of CBMC sample.


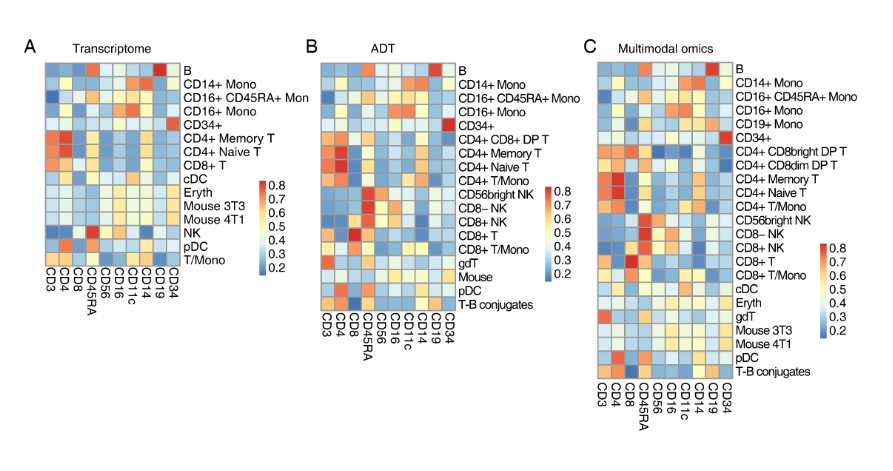


Supplementary Figure 2. Different ADT abundance of each cluster in CBMC sample. Heatmap showing the different ADT abundance of each cluster. The annotation of cell clusters is analyzed by CITEMO using transcriptome modality (A), ADT modality (B) and multimodal omics (C) data, respectively.


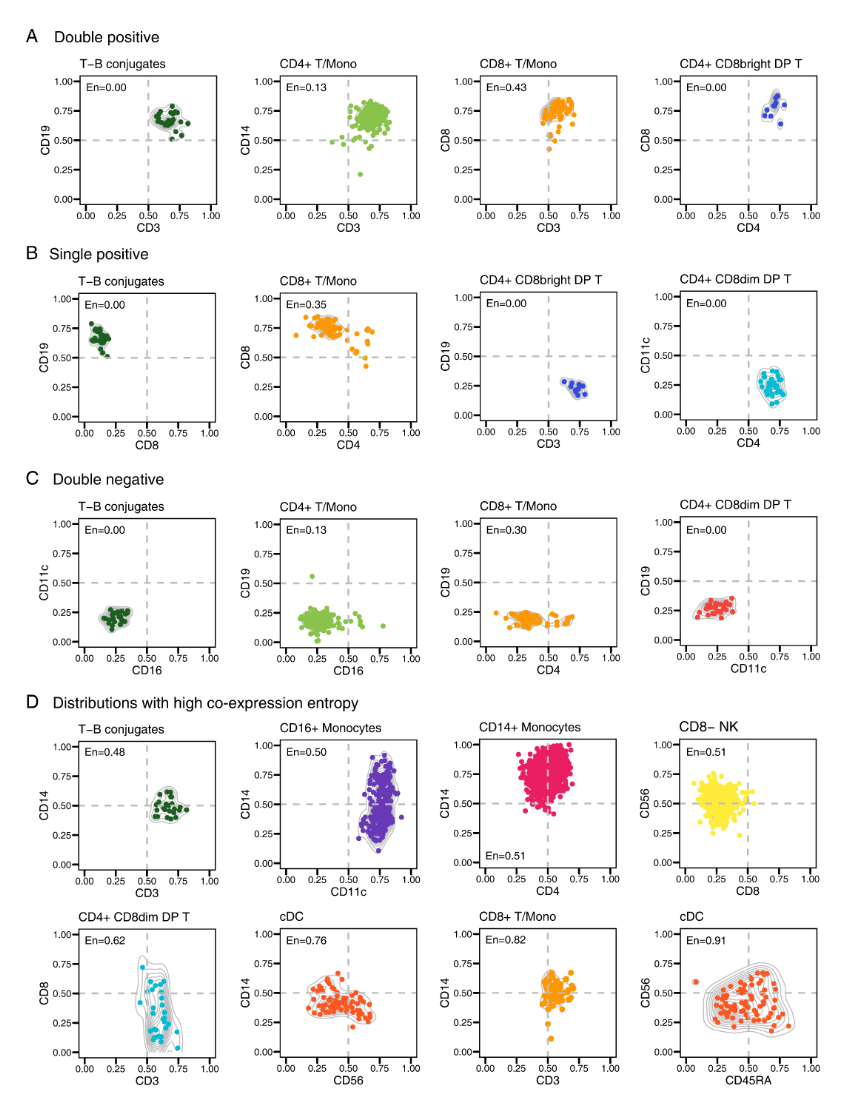


Supplementary Figure 3. Analysis of co-expression entropy in CBMC. (A) Cell clusters with double-positive ADT expression. (B) Cell clusters with single-positive/single-negative expression. (C) Cell clusters with double-negative expression. (D) Co-expression entropy of cell clusters with complex co-expressions.
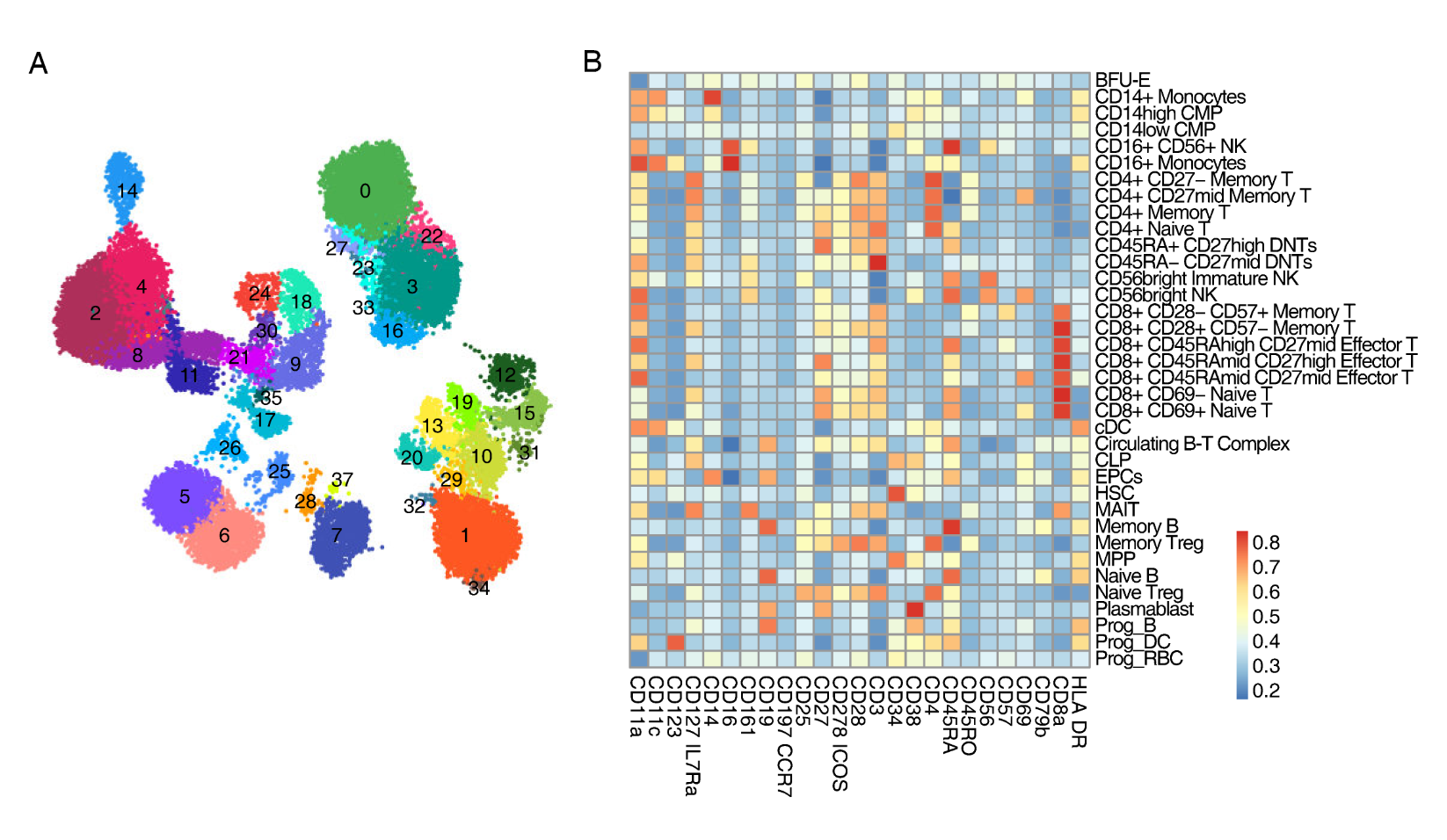


Supplementary Figure 4. Multimodal omics analysis of HBMC sample. (A) UMAP visualizations of clustering results in HBMC sample before manually merge. The annotation of cell clusters is analyzed by CITEMO multimodal omics. (B) Heat map showing the different ADT abundance of each cluster in HBMC sample. The clustering results are analyzed by CITEMO multimodal omics.


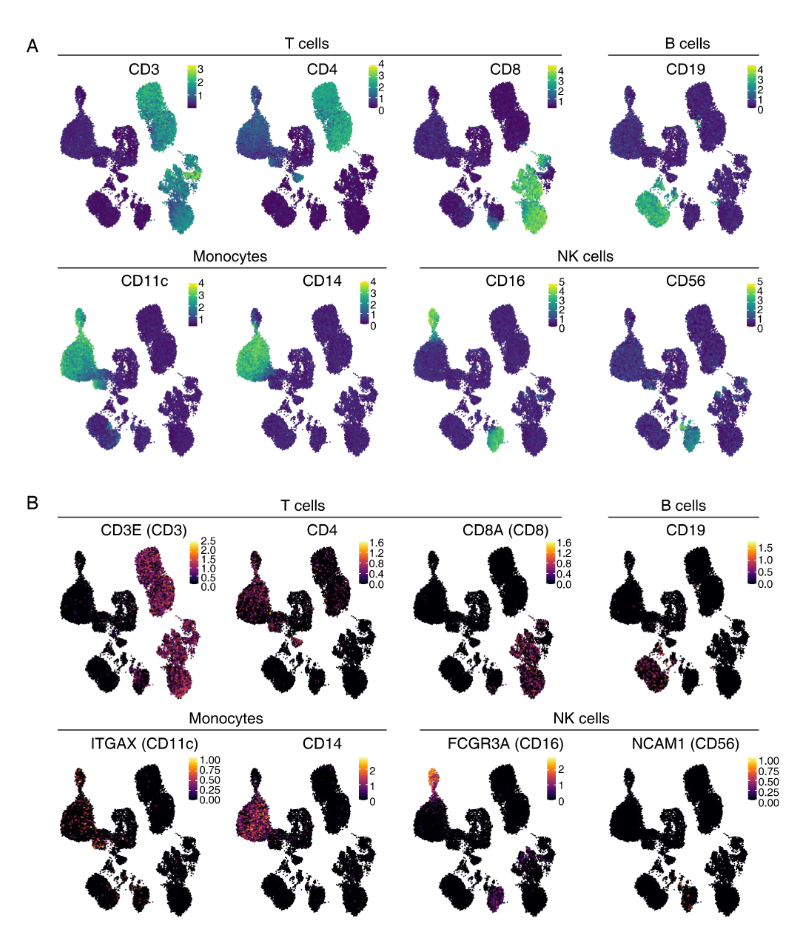


Supplementary Figure 5. Identification of different cluster in HBMC sample. Feature plots showing the ADT abundance (B) and gene expression (A) distributions of common immune cell markers in cell clusters of HBMC sample.


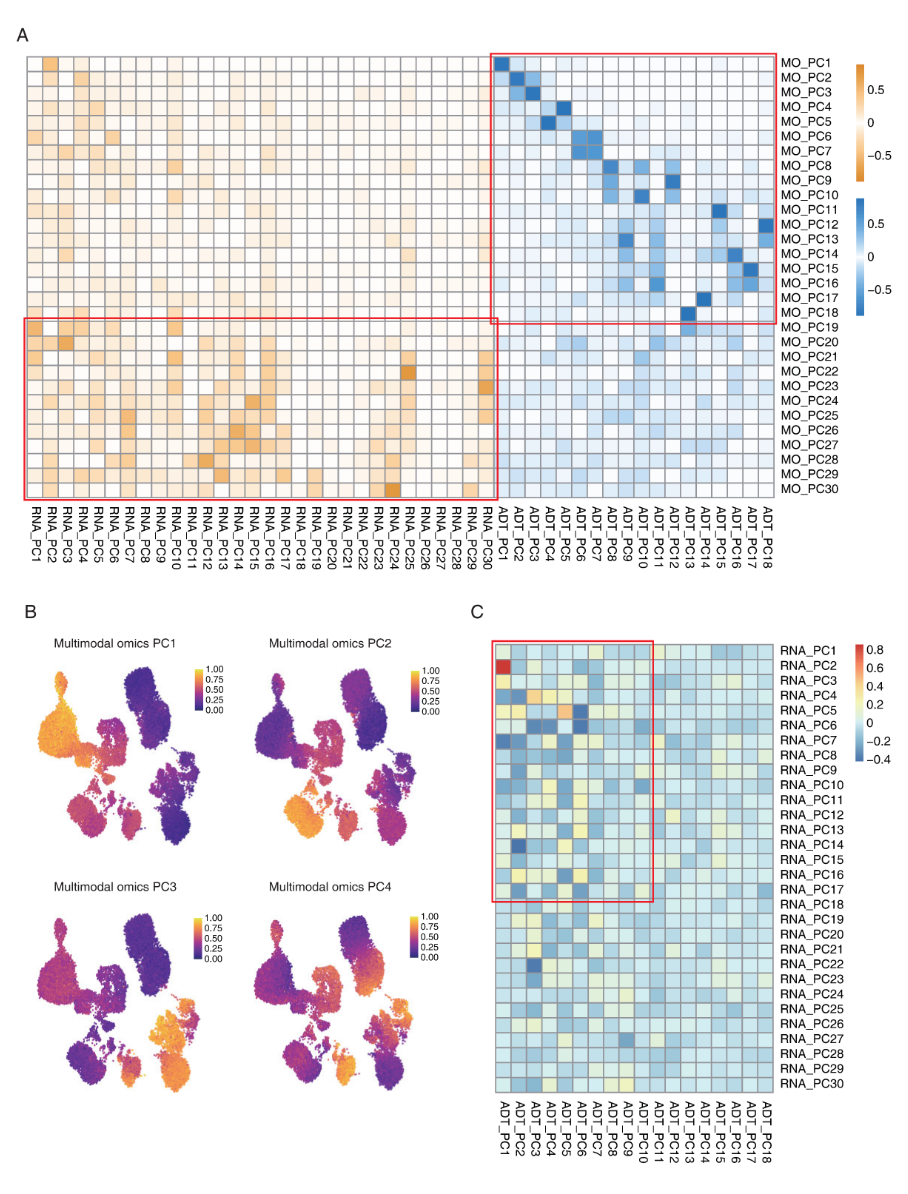


Supplementary Figure 6. The utility of multimodal omics low-dimensional representation in HBMC. (A) Heat map of the features with the largest variance in the low-dimensional representation of CITEMO multimodal omics. The columns represent the multi-modal PCs and the rows represent the features. Features from 1 to 30 come from the PCs with the largest variance in the transcriptome PCs. Features from 31 to 48 come from the PCs with the largest variance in the ADT PCs. (B) UMAP visualizations showing the distribution of PC1 of CITEMO multimodal omics. (C) Spearman correlation of the transcriptome PC and ADT PC.


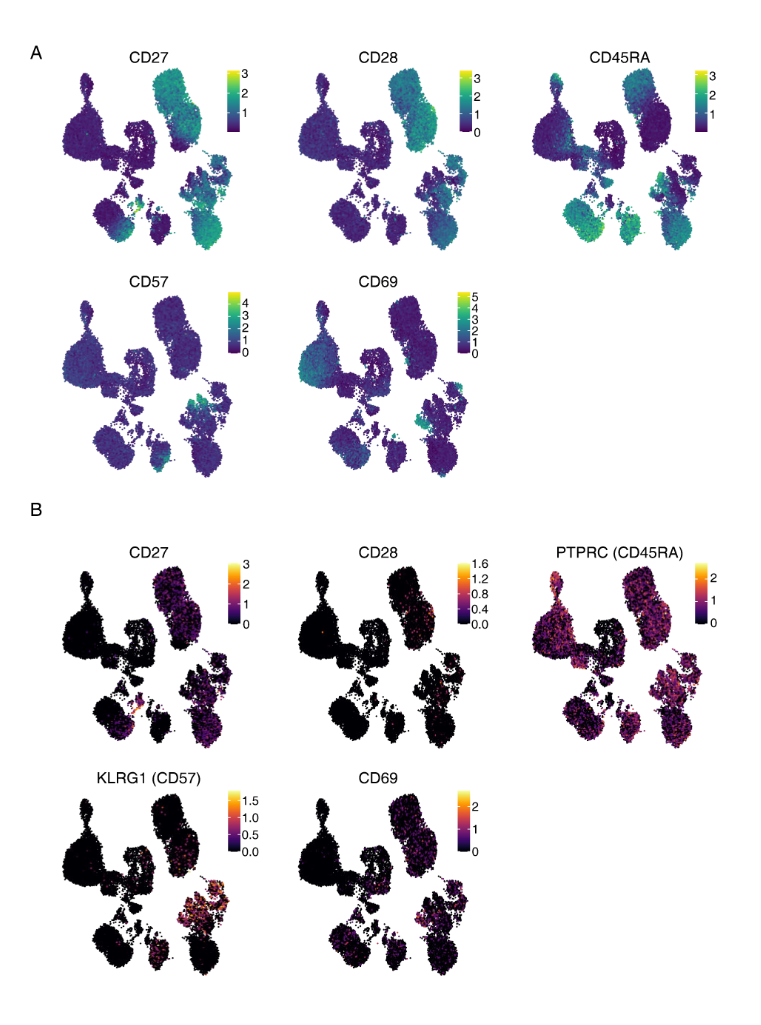


Supplementary Figure 7. Identification of different cell state in HBMC sample. Feature plots showing the ADT abundance (A) and gene expression (B) distributions of cell state markers in cell clusters of HBMC sample.


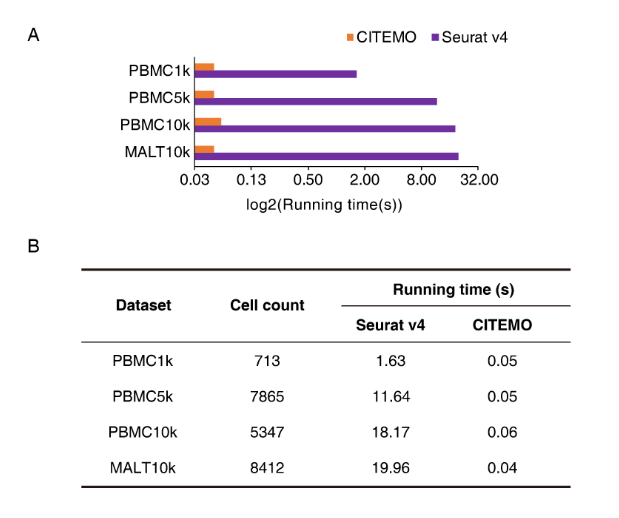


Supplementary Figure 8. Runtime evaluation of integration solutions. Comparison of the time consumed in the data integration step between CITEMO and Seurat v4. (A) The comparison of the logarithm of the running times between CITEMO and Seurat v4 with four different datasets. (B) The running time difference between CITEMO and Seurat v4 with four different datasets.

## Supplementary Table

| **Cell** | **ADT 1** | **ADT 2** | **Double positive ratio** | **ADT1 single positive ratio** | **ADT2 single positive ratio** | **Double negative ratio** | **pearson** | **spearman** | **kendall** | **Distribution entropy** |
| --- | --- | --- | --- | --- | --- | --- | --- | --- | --- | --- |
| T-B conjugates | CD4 | CD19 | 1.00 | 0.00 | 0.00 | 0.00 | 0.05 | -0.05 | -0.02 | 0.00 |
| CD4+ T/Mono | CD8 | CD11c | 0.00 | 0.00 | 0.91 | 0.08 | -0.08 | -0.04 | -0.03 | 0.23 |
| CD8+ T/Mono | CD56 | CD16 | 0.00 | 0.00 | 0.04 | 0.96 | 0.34 | 0.45 | 0.32 | 0.12 |
| CD4+ CD8bright DP T | CD8 | CD11c | 0.00 | 1.00 | 0.00 | 0.00 | -0.61 | -0.69 | -0.49 | 0.00 |
| CD4+ CD8dim DP T | CD4 | CD8 | 0.27 | 0.73 | 0.00 | 0.00 | -0.29 | -0.28 | -0.17 | 0.42 |

Supplementary Table 1. Evaluation of ADT combinations in cell clusters by different methods.
